# Comparison of the effects of fresh leaf and peel extracts of walnut (*Juglans regia L.*) on blood glucose and β-cells of streptozotocin-induced diabetic rats

**Published:** 2012

**Authors:** Somaye Javidanpour, Seyed Reza Fatemi Tabtabaei, Amir Siahpoosh, Hasan Morovati, Ali Shahriari

**Affiliations:** 1 *Department of Basic Sciences, School of Veterinary Medicine, Shahid Chamran University of Ahvaz, Ahvaz, Iran; *; 2 *Department of Pharmacognosy, Faculty of Pharmacy, Ahvaz Jundishapur University of Medical Sciences, Ahvaz, Iran.*

**Keywords:** Diabetes, Walnut leaf, Walnut peel, Hypoglycemia, Insulin

## Abstract

There is some report about the hypoglycemic effect of *Juglans rejia L.* leaf in alloxan induced diabetic rats and hypoglycemic effect of its fruit peel administered intra peritoneally. Thirty male Wistar rats divided into five groups, to evaluate the hypoglycemic and pancreas β-cells regenerative effects of oral methanolic extracts of leaf and fruit peel of walnut. Rats were made diabetic by intravenous (IV) injection of 50 mg kg^-1^ streptozotocin (STZ). Negative control group did not get STZ and any treatment. Positive control, leaf extract, peel extract and insulin groups were treated orally by extract solvent, 200 mg kg^-1^ leaf extract, 200 mg kg^-1^ peel extract and 5 IU kg^-1^ of subcutaneous neutral protamine Hagedorn (NPH) insulin, respectively. Four weeks later, blood was collected for biochemical analysis and pancreases were removed for β-cells counts in histological sections. Diabetes leads to increase of fast blood sugar (FBS) and HbA1c, and decrease of β-cell number and insulin. FBS decreased only in leaf extract group. HbA1c decreased in leaf extract and insulin groups. The β-cells number increased in leaf and peel extract groups. Insulin increased moderately in all treatment groups. We showed the proliferative properties of leaves and peel of *Juglans regia L. *methanolic extract in STZ- induced diabetic rats, which was accompanied by hypoglycemic effect of leaf extract.

## Introduction

Diabetes mellitus is a metabolic disease characterized by hyperglycemia together with impaired metabolism of glucose and other energy-yielding fuels, like lipids and proteins.^1^ These metabolic disorders are the result of insulin deficiency or tissue insulin resistance, or both.^2^ More than 220 million people worldwide suffer from diabetes and this number is likely double by the year of 2030.^3^

Walnut (*Juglans regia*) is a plant in the family Juglandaceae.^4^ Useful parts of walnut tree are leaves, second peel and fleshy part of green fruit and its wood.^5^ Green peel of walnuts fruit, epicarp contains emulsion, glucose, organic acids such as citric acid, malic acid, phosphates, and calcium oxalate. Other materials in its green peel are siaresinolic acid, betulinic acid, daucosterin, α-tetralone, α-glucopyranoside.^6^^,^^7^ Juglon is a 5-Hydroxy 1, 4-naphtoquinone that there is only in green and fresh parts of walnut and it is one of the most important phenolic compounds of walnut leaves and its green peel.^8^^, ^^9^

*Juglans regia L. *has been widely used in Iranian traditional medicine as a remedy for various ailments. Different parts of *Juglans* *regia L. *such as peel, leaves and septum have shown significant hypoglycemic effects.^10^ For example, hydroalcoholic extract of *Juglans* *regia*’s leaves has an anti-hyperglycemic effect in diabetic rats.^11^ Walnut (*Juglans regia L.*) leaves have also been used for treatment of venous insufficiency and hemorrhoidal symptomatology, and for its antidiarrheic, antihelmintic, depurative and astringent properties.^12^^-^^14^ Keratolytic, antifungal, hypotensive, anti-scrofulous and sedative activities have also been described.^15^^,^^16^ Walnut peels, similar to the walnut leaf have some antioxidant,^17^ antifungal,^18^^,^^19^ astringent, and wart liquidator properties used for skin diseases and anemia.^20^

Although there are some reports indicating the regenerative effect of walnut leaves extract on β-cells number and the size of pancreatic islet in alloxan-induced diabetic rats,^21^^, ^^22^ we could not find any report in STZ-induced diabetic animals. However, there were few studies that have examined the effects of IP administration of walnut peel extract in the diabetics,^23^^,^^24^ but there was no study testing the oral effects of walnut peel extract in the diabetic rats. In the present study we have reported the effect of the methanolic extract of leaves and peels of *Juglans regia L.* on the regeneration of β-cells and insulin level in STZ-induced diabetic rats, which provides interesting finding regarding the walnut leaves and the peel properties.

## Materials and Methods

**Extract preparation. **Fresh ripe Walnuts and leaves of *Juglans regia L. *were collected from a garden near Eize (31°48'N, 049°54'E; the South-West of Iran) and were authenticated by Department of Pharmacognosy (Faculty of Pharmacy, Ahvaz Jundishapur University of Medical Sciences). The walnut fruits and leaves were cleaned and the green coat of the walnut fruits peeled. Peels and leaves were powdered by mill. Methanol was added to cover the surface of each powder. Seventy two hr later the solutions were filtered through filter paper. The peel extract was concentrated by rotary evaporator at 40 ˚C and lyophilized to get a powder. To overcome to it's rather insolubility the leaf extract was decanted by chloroform. Then the obtained extract was concentrated by rotary evaporator at 40 ˚C and dried. 

**Animals and specimen collection. **Thirty male Wistar rats weighing 160-200 g were used in this study. Rats were kept in a room with a 12:12 hr light/dark cycle and controlled temperature (22 ± 1 ˚C). Commercial rat pellets and tap water were available *ad libitum*. They were allowed to adapt to the laboratory conditions for one week before the study.^25^ Diabetes was induced by IV injection of STZ (Alexis Corporation, Switzerland; 50 mg kg^-1^) dissolved in 0.1 M citrate buffer (pH=4.5). Negative control group received equal volume of citrate buffer, with no manipulation during the study. One week later, the FBS of negative control and diabetic animals were measured by commercial glucose kit (Parsazmun, Karaj, Iran). Survived rats with marked hyperglycemia (FBS > 300 mg dL^-1^) were selected as diabetic rats, divided into 4 diabetic groups. A solution of distilled water and propylene glycol (3/2: v/v) was used to dissolve the extracts. Positive control group, peel extract group and leaf extract group were fed through a tube with daily administration 2 mg kg^-1^ of extract solvent, 200 mg kg^-1^ of peel extract and 200 mg kg^-1^ of leaf extract,^21^^, ^^26^ respectively. Insulin group treated with daily SC injection of 5 IU kg^-1^ of neutral protamine Hagedorn (NPH) insulin.^27^

Twenty eight days after diabetes confirmation, animals were anesthetized deeply by chloroform (Merck Darmstadt, Germany) vapor and blood was taken from their heart. They were subsequently euthanized under chloroform vapor. Pancreases were fixed in 10% formalin. Blood samples were transferred to EDTA containing tube, 500 μL removed for measurement of glycosylated hemoglobin (HbA1c) and the reminder was centrifuged at 3000 rpm for 15 min to separate plasma for glucose and insulin measurement. 

**Biochemical parameters. **Serum glucose was measured by GOD/POD method (Diagnostic kit: Pars-azmun, Karaj-Iran) using a visible spectrophotometer at 546 nm. Glycated hemoglobin (HbA1c) was determined by diagnostic kit (Costa Brava 30, Barcelona, Spain). Insulin was measured by ultra-sensitive rat ELISA kit (Mercodia, Uppsala-Sweden).

**Tissue processing and **β-**cells count. **Pancreases were fixed in 10% formalin, and 3-5 μm thick sections were prepared by the routine methods and stained with Hematoxylin and Eosin (H&E). Cells with small and dark nuclei considered as β-cells and cells with large and clear nuclei as α-cells. Three slides were prepared form each samples. The β-cells in each slide were counted in three fields with 400× magnification. Thus, each sample of pancreases was counted in 9 fields. Finally, the mean number of β-cells in nine fields was considered as the β-cells number of pancreases in each rat.

**Statistical analysis. **Data were analyzed by SPSS (version 16 for windows, SPSS Inc., Chicago, IL, USA) and expressed as mean ± standard error of mean (SEM). Statistical evaluation was done using one-way analysis of variance (ANOVA) followed by Tukey’s test to analyze the differences between the groups. The data obtained from FBS test were analyzed using paired sample *t*-test. A *p*-value less than 0.05 was considered significant.

## Results

Induction of diabetes increased FBS (*p* < 0.001) and HbA1c (*p* < 0.001) of positive control group (Table 1). The leaf extract significantly decreased glucose level (*p* < 0.05) and HBA1c (*p* = 0.001). There was no significant decrease in FBS of peel extract and insulin groups compared to pretreatment level, but HbA1c significantly decreased in insulin group (*p* < 0.05).

**Fig. 1:**
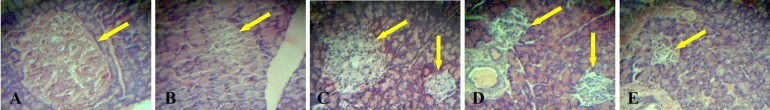
Histological sections of pancreas. **A. **An islet of Langerhans from negative control rat pancreas occupies the center of the field (yellow arrow) **B. **An islet of Langerhans from pancreas of diabetic rat (yellow arrow) **C.** Two islets of Langerhans from pancreas of diabetic rat, treated with walnut peel extract (yellow arrows). **D. **Two islets of Langerhans from pancreas of diabetic rat treated with walnut leaf extract (yellow arrows). **E.** An islet of Langerhans from pancreas of diabetic rat treated with NPH insulin (yellow arrows) (H & E 200×).

Figure 1A indicate a normal islet of Langerhans from negative control group. Beta cell number reduced dramatically in the positive control group (*p* < 0.001) (Fig. 1B) and moderately in insulin group (*p* < 0.05) (Fig. 1E), but it increased in leaf extract (*p* < 0.05) and the peel extract (*p* < 0.001) groups (Figs. 1C and 1D) in comparison to positive control groups. The insulin level reduction was only significant in positive control group (*p* < 0.01). 

**Table 1:** Effect of methanolic extract of leaves and peels of walnut (*Juglans regia L.*) on FBS, HbA1c, β-cell number and plasma insulin level (Mean ± SEM).

**Group**	**FBS (mg dL** ^-1^ **)**		**HbA1c (%)**	**Number of β-ells per counted field**		**Insulin (ng L** ^-1^ **)**
	**Day 0**	**Day 28**				
**Negative control**	93.30 ± 3.60 a	108.10 ± 5.60 a	5.02 ± 0.42 a	88.00 ± 5.00 c	930.00 ± 35.00 b	
**Positive control**	368.20 ± 26.90 bc	405.50 ± 41.50 b	9.72 ± 0.35 c	37.00 ± 5.00 a	224.00 ± 43.00 a	
**Leaf extract**	403.00 ± 62.10 c	283.80 ± 52.20 ab*	6.64 ± 0.68 ab	72.00 ± 7.00 bc	539.00 ± 143.00 ab	
**Peel extract**	421.10 ± 41.90 c	359.60 ± 43.90 b	8.38 ± 0.38 bc	80.00 ± 9.00^ c^	461.00 ± 160.00 ab	
**Insulin**	417.80 ± 61.50 c	372.80 ± 32.70 b	7.49 ± 0.48 b	59.00 ± 5.00 b	535.00 ± 122.00 ab	

a,b,c different letters indicate significant differences between groups (*p* < 0.05).

* indicate significant differences as compared to pre-treatment level (*p *< 0.05).

## Discussion

The hypoglycemic activity of walnut leaves has been reported by previous studies.^10^^, ^^21^^, ^^22^ At the present study, diabetes increased FBS and HbA1c and decreased pancreatic β-cells and plasma insulin. Administration of leaves extract significantly decreased FBS and HbA1c of diabetic animals. Both extracts increased the number of β-cells in diabetic groups, but there was no significant difference in the level of plasma insulin of leaf extract, peel extract and insulin groups in comparison with negative control and diabetic groups. 

Hypoglycemic effect of plants may be due to stimulation of β-cells to produce more insulin,^28^^, ^^29^ increasing glucose metabolism,^30^ improving insulin action and binding carbohydrate^28^ with high level of fiber which interfere with carbohydrate absorption,^31^ presence of insulin-like substances in plants^32^^,^^33^ and/or regenerative effect of plants on pancreatic tissue.^34^ Teimori *et al.* reported that the methanolic extract of walnut leaf inhibited α-glucosidase activity *in vitro* for both maltase and sucrase enzymes.^25^ They did not find any significant increase in the insulin and glut-4 genes expression in the pancreatic and myocardial tissues, respectively.^25^ These findings are in accordance with the recent study, which we did not find any significant increase in the insulin level of leaf extracts treated group. However, Kamyab *et al*. showed 2 hr after treatment by both leaf and ridge hydroalcoholic extracts of walnut, blood glucose and liver gluconeogenic activity have decreased and blood insulin and liver glycogenolysis activity have increased in mild STZ-diabetic mice.^35^ They concluded that walnut was able to lower blood glucose through inhibition of hepatic gluconeogenesis and secretion of pancreatic insulin.^35^ The key compound responsible for inhibitory action of the leaf extract may be phenolic substances, such as gallic acid and caffeoylquinic acid.^17^ A flavonoid fraction extracted from the plant caused a decrease in blood glucose and an increase in β-cells.^36^

Level of HbA1c has been reported to increase in patients with diabetes mellitus.^37^ The level of HbA1c is always monitored as a reliable index of glycemic control in diabetes.^38^ The HbA1c level is proportional to average blood glucose concentration over the previous four weeks to three months.^39^ In our study, treatment of diabetic rats with the leaf extract and insulin significantly decreased the HbA1c level in comparison with the positive control group. Therefore, it was thought the hypoglycemic effect of leaf extract and insulin had begun from the beginning days of the treatments. It was suggested that herbal insulin-like substances in the walnut leaf might be responsible for the hypoglycemic properties of the walnut leaf.^40^ Despite the hypoglycemic effect of insulin and the reduction of HbA1c in the insulin treated group, no change was seen in the initial and final FBS of the insulin group. This is acceptable, because no treatment was done at least from 30 hr before specimen collection. 

Although administration of the peel extract to diabetic rats had no significant effect on FBS and HbA1c level, but it increased the number of β-cells significantly compared to the positive control group. Green peel and leaves of walnut contain antioxidants such as flavonoides.^17^ Recent studies have shown the flavonoids could reduce blood sugar.^10^^,^^41^ The peel of walnut has been used in traditional medicine to reduce the blood glucose in diabetic patients.^10^ Increasing number the β-cells in peel extract group without changes in FBS and HbA1c level might be related to the dysfunctional β-cells unable to produce enough insulin or produce suitable response to glucose elevation. If the duration of the experiment was longer may led to reduction of FBS and HbA1c by increasing β-cell responsiveness and insulin secretion. The leaves and peel of walnut may have some protective and regenerative effect on β-cells. 

In conclusion, according to the results of this study methanolic extract of fresh walnut leaves have significant hypoglycemic properties, which might be related to its insulinomimetic, insulinotropic and/or β-cells regenerative effect of the extract in STZ-diabetic rats. Methanolic extract of fresh walnut peel extract increased β-cells regeneration after 28 days treatment of STZ- diabetic rats. This may be the first report emphasis the proliferative effects of walnut peel on β-cells of diabetic rats and proliferative effects of walnut leaf on STZ treated islets. 
